# Single-qubit reaped quantum state tomography

**DOI:** 10.1038/s41598-022-15089-7

**Published:** 2022-06-29

**Authors:** Mahn-Soo Choi

**Affiliations:** Department of Physics, Korea University, Seoul, 02841 Republic of Korea

**Keywords:** Physics, Quantum physics, Quantum information

## Abstract

Quantum state tomography is the experimental procedure of determining an unknown state. It is not only essential for the verification of resources and processors of quantum information but is also important in its own right with regard to the foundation of quantum mechanics. Standard methods have been elusive for large systems because of the enormous number of observables to be measured and the exponential complexity of data post-processing. Here, we propose a new scheme of quantum state tomography that requires the measurement of only three observables (acting jointly on the system and pointer) regardless of the size of the system. The system is coupled to a “pointer” of single qubit, and the wavefunction of the system is “reaped” onto the pointer upon the measurement of the system. Subsequently, standard two-state tomography on the pointer and classical post-processing are used to reconstruct the quantum state of the system. We also developed an efficient and scalable iterative maximum likelihood algorithm to estimate states from statistically incomplete data.

## Introduction

To develop reliable quantum resources and devices for quantum information processing, it is crucial to verify their actual performance. This is achieved at various levels, such as quantum process tomography^1^ and quantum detector tomography^2,3^, and at the most fundamental level is the quantum state tomography, which is the procedure of experimentally determining an unknown quantum state^4^. Quantum state tomography is of great interest in its own right with regards to the foundation of quantum mechanics as well.

In the standard formulation^5^, quantum state tomography is accomplished by performing repeated measurements of numerous non-commuting observables on many systems prepared in the same states. As a matter of principle, if the set of non-commuting observables is complete and the measurements are repeated infinitely many times, one can build up a comprehensive description of the quantum state by post-processing the measurement statistics^6,7^. It is recapitulated by the three requirements of the standard quantum state tomography: (i) a complete set of observables to be measured (so-called “quorum”), (ii) accurate measurement statistics, and (iii) efficient post-processing. In practice, the requirement of measuring a complete set of observables causes overwhelming experimental obstacles, which affects the other requirements. Technical reasons and other difficulties may prevent some observables from being measured experimentally. For large systems, the number of required observables is exponentially large and places a serious limit on the number of repetitions of measurements (which is finite anyway in reality). Both issues lead to incomplete measurement statistics and/or limited accuracy of measurement statistics. Furthermore, even if reasonably accurate measurement statistics are attained, the complexity of post-processing itself is exponentially high for large systems. To overcome such difficulties in *exact* quantum state tomography, various statistical methods have been developed to estimate quantum states, such as the maximum likelihood estimation^8,9^ and Bayesian estimation^10–12^ methods. Notably, most statistical estimation methods, including the ML and Bayesian approaches, are highly nonlinear procedures and generally suffer from high complexity for large systems.

Here, we propose a new quantum state tomography scheme that requires the measurement of only three *observables* regardless of the system size^13^. In this scheme, the system is coupled to a “pointer” of a single two-level quantum system (i.e., “qubit”), and the wavefunction of the system is “reaped” onto the pointer upon the measurement of a single observable on the system. The subsequent standard quantum state tomography on the pointer and classical post-processing reconstruct the quantum state of the system, where the classical post-processing requires matrix inversion. We refer to this scheme as *single-qubit reaped* (or *pointer-reaped*) quantum state tomography. We have also developed an iterative maximum likelihood (ML) estimation algorithm that is adaptable to the single-qubit reaped scheme. The iterative ML estimation algorithm is demonstrated by numerical simulations with several interesting quantum states, such as the GHZ, W, and Dicke states. Furthermore, by matrix product state (MPS) representations, the iterative ML algorithm is scalable and provides an efficient method to obtain MPS estimates for the mixed states of large systems. The MPS pure state estimate for the mixed state determines the lower bound of the fidelity between the pure and mixed states and can be used to experimentally verify the purity of the laboratory-generated states^14^.

## Results

### Exact tomography

**Figure 1:**
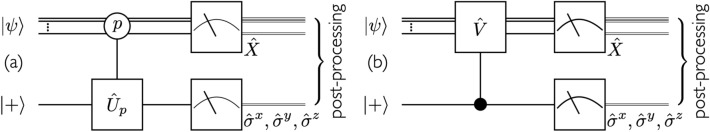
Two equivalent schematics of the single-qubit reaped quantum state tomography. (**a**) The system–pointer interaction is described by the *p*-dependent conditional phase shift $\hat{U}_p:=\mathinner {|{\textstyle 0}\rangle }\mathinner {\langle {\textstyle 0}|}+e^{ip\theta }\mathinner {|{\textstyle 1}\rangle }\mathinner {\langle {\textstyle 1}|}$ on the pointer. (**b**) The system–pointer interaction is regarded as the pointer-controlled unitary operator $\hat{V}:=e^{i\theta \hat{P}}$ on the system. The measurement on the system measures the eigenvalues *x* of the observable $\hat{X}:=\sum _xx\mathinner {|{\textstyle x}\rangle }\mathinner {\langle {\textstyle x}|}$ whereas the measurement on the pointer measures the eigenvalues of the Pauli operators $\hat{\sigma }^x$, $\hat{\sigma }^y$, or $\hat{\sigma }^z$.

Consider a system of *n* particles, each of which has dimension *d*, such that the total dimension of the system is $N := d^n.$ Let ${\textstyle \{\mathinner {|{\textstyle x}\rangle }|x=0,\ldots ,N-1\}}$ be the computational basis of the Hilbert space. Suppose that we have an ensemble of such systems, identically prepared in the unknown state $\mathinner {|{\textstyle \psi }\rangle }= \sum _{x=0}^{N-1}\mathinner {|{\textstyle x}\rangle }\psi _x,$ with the “wavefunction” $\psi _x\in \mathbb {C}$, where $\mathbb {C}$ is the set of complex numbers. We assume that $\psi _0\ne 0$ without a loss of generality (a physical state cannot be a null vector). Our proposed scheme is illustrated in the two equivalent quantum circuits in Fig. 1. We discuss these procedures in the following order:

First, we select a qubit as the “pointer”. The pointer plays a central role in the proposed scheme. Initially, we prepare the pointer in the state $\mathinner {|{\textstyle +}\rangle }:=(\mathinner {|{\textstyle 0}\rangle }+\mathinner {|{\textstyle 1}\rangle })/\sqrt{2}$, where $\mathinner {|{\textstyle 0}\rangle }$ and $\mathinner {|{\textstyle 1}\rangle }$ are the computational basis states of the pointer such that the initial state of the system plus pointer is given by $\mathinner {|{\textstyle \Psi }\rangle }= \sum _x\mathinner {|{\textstyle x}\rangle }\psi _x\otimes \mathinner {|{\textstyle +}\rangle }$.

Next, we couple the system and pointer for a certain time, which is assumed to be sufficiently short compared to the typical time scales of the system and pointer. This interaction can be described by a unitary operator of the form^15^ $\hat{U}_\mathrm {int} = \exp \left( i\theta \hat{P}\otimes \mathinner {|{\textstyle 1}\rangle }\mathinner {\langle {\textstyle 1}|}\right) ,$ where $\hat{P}$ is an observable of the system. For the sake of physical implementation in actual experiments, one can take two different but equivalent views of $\hat{U}_\mathrm {int}$. One can represent $\hat{U}_\mathrm {int}$ with the phase shift on the pointer *conditioned* on the system observable $\hat{P}$. To observe this more explicitly, let $\mathinner {|{\textstyle p}\rangle }$ be the eigenstate of the observable $\hat{P}$ belonging to the eigenvalue *p* and rewrite $\hat{U}_\mathrm {int}$ as $\hat{U}_\mathrm {int} = \sum _p\mathinner {|{\textstyle p}\rangle }\mathinner {\langle {\textstyle p}|}\otimes \hat{U}_p$ with the *p*-dependent phase shift $\hat{U}_p := \mathinner {|{\textstyle 0}\rangle }\mathinner {\langle {\textstyle 0}|} + e^{ip\theta }\mathinner {|{\textstyle 1}\rangle }\mathinner {\langle {\textstyle 1}|}$ on the pointer. This interpretation is depicted in the quantum circuit representation in Fig. 1a and is analogous to the conventional von Neumann picture of the measurement of the observable $\hat{P}$. One important difference is that the pointer here is only of two dimensions and is insufficient to directly discriminate the *N* eigenvalues, *p*, of $\hat{P}$. On the other hand, noting that $\hat{U}_\mathrm {int}=\hat{I}\otimes \mathinner {|{\textstyle 0}\rangle }\mathinner {\langle {\textstyle 0}|}+\hat{V}\otimes \mathinner {|{\textstyle 1}\rangle }\mathinner {\langle {\textstyle 1}|}$ with $\hat{I}$ being the identity operator and $\hat{V}:=e^{i\theta \hat{P}}$, one can regard it as a pointer-controlled unitary operator $\hat{V}$ acting on the system. This picture is illustrated in the quantum circuit in Fig. 1b and is analogous to the quantum phase estimation circuit for a unitary transformation ($\hat{V}$ in the present case)^16^. Throughout this paper, we will mainly consider the latter interpretation for convenience. After the unitary interaction, the total state becomes

1 $$\begin{aligned} \hat{U}_\mathrm {int}\mathinner {|{\textstyle \Psi }\rangle }= \sum _{xy}\mathinner {|{\textstyle x}\rangle }\otimes \frac{\mathinner {|{\textstyle 0}\rangle }\delta _{xy}\psi _y + \mathinner {|{\textstyle 1}\rangle }V_{xy}\psi _y}{\sqrt{2}}, \end{aligned}$$

where *V* is the matrix representation of $\hat{V}$ in the computational basis,

2 $$\begin{aligned} V_{xy} := \mathinner {\langle {\textstyle x|\hat{V}|y}\rangle } = \sum _{p}\mathinner {\langle {\textstyle x|p}\rangle }e^{ip\theta }\mathinner {\langle {\textstyle p|y}\rangle }. \end{aligned}$$

We then measure the eigenvalues of the observable $\hat{X}:=\sum _xx\mathinner {|{\textstyle x}\rangle }\mathinner {\langle {\textstyle x}|}$ in the system. When the measurement outcome is *x*, the (unnormalized) pointer state is reduced to

3 $$\begin{aligned} \mathinner {|{\textstyle \phi _x}\rangle } = \mathinner {|{\textstyle 0}\rangle }\psi _x + \mathinner {|{\textstyle 1}\rangle }\sum _{y}V_{xy}\psi _y. \end{aligned}$$

Equation (3) reveals the key idea of the proposed scheme: the wavefunction $\psi _x$ appears in the two expansion coefficients and can be determined by the standard quantum state tomography by measuring three independent observables, that is, the Pauli operators $\hat{\sigma }^x$, $\hat{\sigma }^y$, and $\hat{\sigma }^z$ in the pointer. One tricky point is that naive two-state tomography does not fix the overall phase, which is necessary to fix the relative phases of $\psi _x$ for different values of *x*. We now provide a careful tomographic reconstruction procedure [see Eq. (6)] that is not hindered by this tricky issue.

Physically, the two-step procedure for the measurement of $\hat{X}$ on the system and the subsequent quantum state tomography on the pointer is equivalent to the measurement of the eigenvalues of three observables, $\hat{X}\otimes \hat{\sigma }^z$, $\hat{X}\otimes \hat{\sigma }^x$, and $\hat{X}\otimes \hat{\sigma }^y$. For the purpose of mathematical analysis of measurement outcomes and maximum likelihood estimation process (see below), it is convenient to describe the measurements using the projective POVM elements

4 $$\begin{aligned} \hat{\Pi }_{x,m} := \frac{1}{3}\hat{\Pi }_x\otimes \hat{\Pi }_m, \end{aligned}$$

where $\hat{\Pi }_x=\mathinner {|{\textstyle x}\rangle }\mathinner {\langle {\textstyle x}|}$, $\hat{\Pi }_m=\mathinner {|{\textstyle m}\rangle }\mathinner {\langle {\textstyle m}|}$, and the index $m\in \mathscr {M}:={\textstyle \{0,1,+,-,L,R\}}$ refers to the eigenstates $\mathinner {|{\textstyle m}\rangle }=\mathinner {|{\textstyle 0}\rangle },\mathinner {|{\textstyle 1}\rangle },\mathinner {|{\textstyle +}\rangle },\mathinner {|{\textstyle -}\rangle },\mathinner {|{\textstyle L}\rangle },\mathinner {|{\textstyle R}\rangle }$ of the Pauli operators $\hat{\sigma }^z$, $\hat{\sigma }^x$, and $\hat{\sigma }^y$, respectively. The joint probabilities $P_{x,m} =\mathinner {\langle {\textstyle \Psi }|}\hat{U}_\mathrm {int}^\dag \hat{\Pi }_{x,m}\hat{U}_\mathrm {int}\mathinner {|{\textstyle \Psi }\rangle }$ determine the ratio between the two coefficients,

5 $$\begin{aligned} \frac{1}{\psi _x}\sum _{y=0}^{N-1}V_{xy}\psi _y = \sqrt{\frac{P_{x,1}}{P_{x,0}}}e^{i\varphi _x}, \end{aligned}$$

where $\varphi _x := \arg [(P_{x,+}-P_{x,-}) + i(P_{x,L}-P_{x,R})].$ Owing to the normalization constraint, the *N* relations in Eq. (5) are not independent of each other. Instead of directly imposing the normalization constraint, one can just determine the ratio $\psi _x/\psi _0$. This casts the relation (5) to the following set of $(N-1)$ linear equations

6 $$\begin{aligned} \sum _{y=1}^{N-1}\left\{ \sqrt{P_{x,1}}e^{i\varphi _x}\delta _{xy} - \sqrt{P_{x,0}}V_{xy} \right\} \left( \frac{\psi _y}{\psi _0}\right) = \sqrt{P_{x,0}}V_{x0} \end{aligned}$$

for $x=1,\ldots ,N-1$. Given the experimentally determined measurement statistics $P_{x,m}$, solving the linear equations yields the wavefunction $\psi _x$ (up to normalization). There are several dangerous cases in which Eq. (6) cannot provide a unique solution. Avoiding or overcoming them is addressed in “Methods”.

One simple example is to select the local basis $\mathinner {|{\textstyle x}\rangle }$ such that $\mathinner {\langle {\textstyle x|p}\rangle } = N^{-1/2}e^{2\pi {i} xk_p/N},$ where $k_p$ is the index of *p* when the eigenvalues are arranged in an ordered sequence. The computational basis $\mathinner {|{\textstyle x}\rangle }$ and the eigenstates $\mathinner {|{\textstyle p}\rangle }$ of $\hat{P}$ are related by the quantum Fourier transform^17^. For a system consisting of qubits ($d=2$), another valuable example is the system operator of the form $\hat{P}= \sum _{j=1}^n\hat{\tau }_j^x,$ where $\hat{\tau }_j^x:=(\mathinner {|{\textstyle 0}\rangle }\mathinner {\langle {\textstyle 1}|}+\mathinner {|{\textstyle 1}\rangle }\mathinner {\langle {\textstyle 0}|})_j$ denotes the Pauli operator acting on the *j*th qubit. This leads to a pointer-controlled unitary operator

7 $$\begin{aligned} \hat{V}= e^{i\theta \hat{P}} = \begin{bmatrix} \cos \theta &{} i\sin \theta \\ i\sin \theta &{} \cos \theta \end{bmatrix}^{\otimes n} \quad (0<\theta <\pi /2) \end{aligned}$$

In this case, $\mathinner {|{\textstyle x}\rangle }$ and $\mathinner {|{\textstyle p}\rangle }$ are related to each other via the local Hadamard gates,

8 $$\begin{aligned} \left[ \mathinner {\langle {\textstyle x|p}\rangle }\right] _{x,p=0,1,\cdots ,2^n-1} = H^{\otimes n} \end{aligned}$$

with

9 $$\begin{aligned} H := \frac{1}{\sqrt{2}} \begin{bmatrix} 1 &{} 1 \\ 1 &{} -1 \end{bmatrix}. \end{aligned}$$

### Maximum likelihood estimation algorithm

Above, we have shown that, as a matter of principle, the single-qubit reaped scheme can successfully reconstruct quantum states. It assumes an idealistic situation where the probability distribution $P_{x,m}$ corresponding to the POVM elements $\hat{\Pi }_{x,m}$ can be inferred from measurements. It is possible only when the measurements are repeated infinitely many times, apart from other technical imperfections; finite repetitions give rise to statistical errors in the inferred probabilities $P_{x,m}$. Obviously, the statistical errors become more severe as the system size *n* increases; recall the number $6d^n$ of possible measurement outcomes (*x*, *m*). A popular method to overcome such an issue is to follow the maximum likelihood (ML) principle and seek the state that is most “likely” given the experimental observations rather than the actual (and impossible-to-infer) wavefunction^6–9,18^. In this section, we develop an iterative ML algorithm that can be combined with the single-qubit reaping scheme discussed above. We note controversies about the physically proper estimation of quantum states from the experimental data^11,18^, and it would be valuable to develop other statistical methods, such as Bayesian approaches, that are adaptable to the present tomography scheme.

Consider an ensemble of *F* systems. Let $F_{x,m}$ be the number of experimental observations corresponding to the POVM element $\hat{\Pi }_{x,m}$, such that $F = \sum _{x,m}F_{x,m}.$ The ideal situation corresponds to the limit $F\rightarrow \infty$, where the relative frequency $F_{x,m}/F$ gives the true probability $P_{x,m}$. For finite size ($F<\infty$), $F_{x,m}/F$ only estimates $P_{x,m}$ approximately. The observation statistics are governed by a multinomial distribution

10 $$\begin{aligned} \mathscr {L}= F!\prod _{x}\prod _{m\in \mathscr {M}}\frac{(P_{x,m})^{F_{x,m}}}{F_{x,m}!}, \end{aligned}$$

where

11 $$\begin{aligned} P_{x,m} =\mathinner {\langle {\textstyle \Psi }|}\hat{U}_\mathrm {int}^\dag \hat{\Pi }_{x,m}\hat{U}_\mathrm {int}\mathinner {|{\textstyle \Psi }\rangle } \end{aligned}$$

is the probability of obtaining the result (*x*, *m*) on the condition that the system plus pointer is prepared in the state $\mathinner {|{\textstyle \Psi }\rangle }=\mathinner {|{\textstyle \psi }\rangle }\otimes \mathinner {|{\textstyle +}\rangle }.$

We use the multinomial distribution $\mathscr {L}$ as the likelihood function. Generally, the likelihood function should depend on the specific measurement apparatus and other experimental conditions. Here, we focus on the generic effects on statistical error, putting aside specific technical issues. The ML approach maximizes

12 $$\begin{aligned} \log \mathscr {L}= \sum _{x}\sum _{m\in \mathscr {M}}F_{x,m}\log {P_{x,m}} \end{aligned}$$

(up to irrelevant terms) over all possible states $\mathinner {|{\textstyle \psi }\rangle }$ of the system with the normalization constraint. The wavefunction $\bar{\psi }_x$ that maximizes the likelihood function satisfies the extremal equation (see “Methods” for details)

13 $$\begin{aligned} \sum _yW_{xy}\bar{\psi }_y = \bar{\psi }_x, \end{aligned}$$

where the matrix *W* is defined by

14 $$\begin{aligned} W_{xy} := \mathinner {\langle {\textstyle 0|\hat{R}_x|0}\rangle }\delta _{xz} + \mathinner {\langle {\textstyle 0|\hat{R}_x|1}\rangle }V_{xy} + V^\dag _{xy}\mathinner {\langle {\textstyle 1|\hat{R}_y|0}\rangle } + \sum _zV^\dag _{xz}\mathinner {\langle {\textstyle 1|\hat{R}_z|1}\rangle }V_{zy} \end{aligned}$$

and the *x*-dependent operator $\hat{R}_x$ on the pointer by

15 $$\begin{aligned} \hat{R}_x := \sum _{m\in \mathscr {M}} \frac{F_{x,m}}{P_{x,m}} \mathinner {|{\textstyle m}\rangle }\mathinner {\langle {\textstyle m}|}. \end{aligned}$$

Formally, $\hat{R}_x$ is reminiscent of a similar operator (denoted by $\hat{R}$) that appears in the iterative maximization algorithm adapted to the standard tomography scheme^9^. In our case, $\hat{R}_x$ acts on the pointer and not on the system itself. In an ideal experiment where $F\rightarrow \infty$, the true wavefunction indeed gives the extremum solution, $\bar{\psi }_x=\psi _x$, as $\hat{R}_x=\hat{I}$. In a realistic experiment with a finite-size ensemble ($F<\infty$), in general $\bar{\psi }_x\ne \psi _x$, but $\bar{\psi }_x$ is simply the wavefunction most likely for the given measurements data.

**Figure 2:**
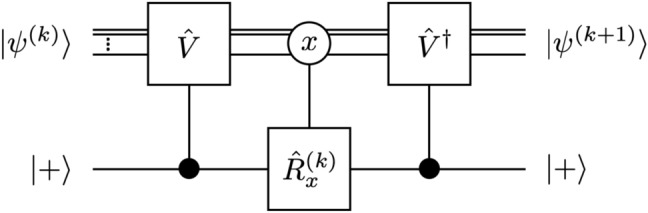
Quantum circuit interpretation of the maximum likelihood iteration. The solid dot indicates the “controlled”-$\hat{V}$ (or $\hat{V}^\dag$) acting only when the pointer is in the state $\mathinner {|{\textstyle 1}\rangle }$ whereas the open circle indicates “conditional”-$\hat{R}_x$ on the pointer conditioned on the state $\mathinner {|{\textstyle x}\rangle }$ of the system. Despite the quantum circuit interpretation, the iteration procedure is *not* linear as the operator $\hat{R}_x[\psi ^{(k)}]$ depends on the trial state $\mathinner {|{\textstyle \psi ^{(k)}}\rangle }$.

It should be noted that the operator $\hat{R}_x$ depends functionally on the state $\mathinner {|{\textstyle \psi }\rangle }$ through the probability $P_{x,m}$, and hence the extremum Eq. (13) is *nonlinear*. Solving such a nonlinear equation is unviable, particularly for large systems (involving a large number of variables $\psi _x$). Instead, we have developed an iterative algorithm^9,18–20^. First, we need to choose an initial trial wavefunction. From the pointer state $\mathinner {|{\textstyle \phi _x}\rangle }$ in Eq. (3) upon the measurement readout *x*, it follows that the probability $P_{x,0}$ is directly proportional to $|\psi _x|^2$. This implies that $\mathinner {|{\textstyle \psi ^{(0)}}\rangle } \propto \sum _x\mathinner {|{\textstyle x}\rangle }\sqrt{F_{x,0}/F}$ is a reasonable choice. At each iterative step *k*, the wavefunction $\mathinner {|{\textstyle \psi ^{(k)}}\rangle }$ is updated using the mapping

16 $$\begin{aligned} \hat{W}[\psi ^{(k)}]\mathinner {|{\textstyle \psi ^{(k)}}\rangle } = \mathinner {|{\textstyle \psi ^{(k+1)}}\rangle }, \end{aligned}$$

where the iteration generator $\hat{W}:=\sum _{xy}W_{xy}\mathinner {|{\textstyle x}\rangle }\mathinner {\langle {\textstyle y}|}$ is constructed from the matrix *W* in Eq. (14). Interestingly, the iteration procedure can be represented by the quantum circuit shown in Fig. 2, which illustrates the crucial role of the pointer from another perspective. The quantum circuit itself is not advantageous when one evaluates the iterations directly. However, as we will observe later, it clearly reveals the simple mathematical structure of the iteration generator $\hat{W}$, which permits the scalability of the iterative algorithm.

**Figure 3:**
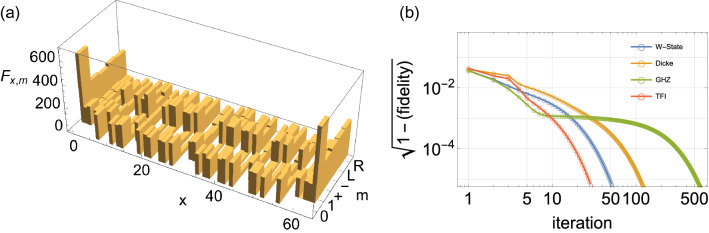
(**a**) Relative frequencies $F_{x,m}$ of the measurement readouts (*x*, *m*) from the simulation with an ensemble of 24,000 systems in the symmetric Dicke state with six qubits ($F=24,000$, $n=6$, $d=2$). (**b**) Convergence behaviors of the iterative maximization procedure for different system states (the W state, Dicke state, GHZ state, and the ground state of the transverse field Ising model in the ordered phase) exhibited by the fidelity between the states from consecutive iterations. For all the four cases, the fidelities between the resulting states and the true wavefunctions, respectively, are better than 0.99.

The convergence of no iterative ML algorithm has been analytically proven^18^. However, in standard ML approaches^21,22^, numerical tests have demonstrated convergence for physically interesting states, and a diluted iterative algorithm is available when the convergence is critical^18^. Here, we demonstrate the algorithm numerically using several examples for a system of six qubits ($n=6$ and $d=2$). The first example is the symmetric Dicke state $\mathinner {|{\textstyle \psi }\rangle }= \sum '\mathinner {|{\textstyle 000111}\rangle }/\sqrt{20},$ where $\sum '$ refers to the summation over all permutations of the qubits. We simulated the measurements for an ensemble of 24,000 systems ($F=24,000$) all prepared in the same state $\mathinner {|{\textstyle \psi }\rangle }$. The resulting relative frequencies, $F_{x,m}$, of the measurement readouts (*x*, *m*) are shown in Fig. 3a. We then obtained the ML estimate $\mathinner {|{\textstyle \psi ^{(500)}}\rangle }$ for the measurement data ($F_{x,m}$) through 500 iterations in accordance with (16). As shown in Fig. 3 (b, blue curve), the *infidelity* between the states from consecutive iterations was already less than $10^{-5}$ after 150 iterations. The fidelity, $\left| \mathinner {\langle {\textstyle \psi ^{(200)}|\psi }\rangle }\right| ^2,$ with the true wavefunction is larger then 0.997.

We performed similar simulations and made the ML estimates for the simulation results for the W-state $\mathinner {|{\textstyle \psi }\rangle }=(\mathinner {|{\textstyle 10 00 00}\rangle }+\mathinner {|{\textstyle 01 00 00}\rangle }+\cdots +\mathinner {|{\textstyle 00 00 01}\rangle })/\sqrt{6},$ the GHZ state $\mathinner {|{\textstyle \psi }\rangle }=(\mathinner {|{\textstyle 000000}\rangle }+\mathinner {|{\textstyle 111111}\rangle })/\sqrt{2},$ and the ground state of the transverse-field Ising model in the ordered phase. Figure 3b corroborates the excellent convergence for all those cases. The fidelities between the ML estimates and the respective true wavefunctions were also as good as 0.99 or larger.

### Scalability and mixed states

Each ML iteration in Eq. (16) involves the multiplication of exponentially large matrices and vectors, and the computational cost of many iterations for the desired accuracy may still be high for large systems. This can be overcome by means of matrix product state (MPS) and matrix product operator (MPO) representations (see “Methods”). We first examine the quantum circuit shown in Fig. 2 more closely to better understand the MPO structure of the iteration generator, $\hat{W}$. Let $\hat{W}_\mathrm {tot}$ be the extended operator acting on the system and pointer, which results in $\hat{W}=\mathinner {\langle {\textstyle +|\hat{W}_\mathrm {tot}|+}\rangle }$ when averaging over the pointer with the state $\mathinner {|{\textstyle +}\rangle }$. $\hat{W}_\mathrm {tot}$ consists of the controlled-unitary operator $\hat{I}\otimes \mathinner {|{\textstyle 0}\rangle }\mathinner {\langle {\textstyle 0}|}+\hat{V}\otimes \mathinner {|{\textstyle 1}\rangle }\mathinner {\langle {\textstyle 1}|}$ and the conditional-unitary operator $\sum _x\mathinner {|{\textstyle x}\rangle }\mathinner {\langle {\textstyle x}|}\otimes \hat{R}_x[\psi ^{(k)}].$

The former is an MPO with a bond dimension of 2 when the coupling observable $\hat{P}$ (and hence $\hat{V}$) is local [Eq. (7) is an example]. The latter is also an MPO with a finite bond dimension provided that the input state $\mathinner {|{\textstyle \psi ^{(k)}}\rangle }$ is an MPS with a finite bond dimension because an MPS only has finite correlations^23,24^; see “Methods”. Therefore, $\hat{W}_\mathrm {tot}$, the product of three MPOs, should be an MPO with a finite bond dimension, and so is $\hat{W}$ as it corresponds to a partial trace of an MPO. Currently, the operation of an MPO on an MPS can be efficiently evaluated^23,24^. In summary, if the laboratory states are MPS, the iteration generator is represented by an MPO, and the ML iterations in Eq. (16) can be updated efficiently. Recently, a formally similar iterative algorithm (from a different tomography scheme) powered by MPO and MPS representations has been demonstrated in detail^22^.

Because only a polynomial number of parameters is required for the MPS representations, they span only a small portion of the entire Hilbert space. However, it is well known that many states relevant to quantum information processing, condensed matter physics, and other areas of physics exist in the MPS form. The ground states of the strongly correlated many-body Hamiltonians as well as the cluster states are notable examples.

Moreover, as was pointed out recently^14^, the tomographic estimation of MPS pure states is valuable even when the system is in a mixed state. That is, it allows us to determine a lower bound on the fidelity between the pure state estimate and mixed states compatible with the experimental observations, thereby certifying the purity of the laboratory state via experiments. A scalable ML method has been proposed to directly reconstruct mixed states via local measurements^21,22^, assuming that the states are close to a MPS. For their method, however, experimenters are required to measure many non-commuting observables whereas our scheme requires the measurement of only three observables $\hat{X}\otimes \hat{\sigma }^x$, $\hat{X}\otimes \hat{\sigma }^y,$ and $\hat{X}\otimes \hat{\sigma }^z,$ regardless of the system size^13^.

## Discussion

A seemingly similar idea to couple the system with an ancillary system and measure only one observable (over the entire system plus ancilla) has been previously proposed^25^; this is the so-called ancilla-assisted quantum state tomography and has been demonstrated in recent experiments^26,27^. However, their scheme required the ancilla to be as large as or even larger than the system (one obvious advantage is that it can directly estimate the density matrix of the system). Moreover, no ML algorithm has been developed for their scheme.

The convergence of the ML iterations varies for different states. For example, it is noted in Fig. 3b that the convergence of the ML iterations is slower for the GHZ state (approximately 500 iterations are required for similar accuracy) than for other states. Recalling the massive and long distance entanglement in the GHZ state, this fact raises an interesting question about the relation between the convergence behavior of our ML iterations and the properties (such as multi-partite entanglement) of the state. We leave the relation as an inspiring open question for future works.

## Methods

### State-reconstruction equation

Here, we derive the state-reconstruction Eq. (5). We begin with the (unnormalized) pointer state in Eq. (1)

17 $$\begin{aligned} \mathinner {|{\textstyle \phi _x}\rangle } = \mathinner {|{\textstyle 0}\rangle }\alpha _x + \mathinner {|{\textstyle 1}\rangle }\beta _x, \end{aligned}$$

where we have defined $\alpha _x:=\psi _x$ and $\beta _x:=\sum _yV_{xy}\psi _y$ for notational simplicity. We want to express the ratio $\beta _x/\alpha _x$ in terms of the joint probabilities $P_{x,m}$. The joint probabilities satisfy the following relationship:

18a $$\begin{aligned} P_{x,0}&= |\alpha _x|^2 , \end{aligned}$$

18b $$\begin{aligned} P_{x,1}&= |\beta _x|^2 ,\end{aligned}$$

18c $$\begin{aligned} P_{x,+} - P_{x,-}&= \alpha _x^*\beta _x + \alpha _x\beta _x^* ,\end{aligned}$$

18d $$\begin{aligned} i(P_{x,L} - P_{x,R})&= \alpha _x^*\beta _x - \alpha _x\beta _x^*. \end{aligned}$$

Using the last two relations, one can obtain

19 $$\begin{aligned} P_{x,+}-P_{x,-} + i(P_{x,L}-P_{x,R}) = 2\alpha _x^*\beta _x. \end{aligned}$$

This implies that the relative phase between $\alpha _x$ and $\beta _x$, which is the essential part for quantum coherence effects, can be extracted by combining the join probabilities on the left-hand side. More explicitly, we express it as

20 $$\begin{aligned} \varphi _x := \arg \left[ P_{x,+}-P_{x,-} + i(P_{x,L}-P_{x,R})\right] , \end{aligned}$$

and observe that

21 $$\begin{aligned} e^{i\varphi _x} = \frac{ P_{x,+}-P_{x,-} + i(P_{x,L}-P_{x,R}) }{2|\alpha _x\beta _x|} = \frac{\alpha _x^*}{|\alpha _x|}\frac{\beta _x}{|\beta _x|} = \frac{\beta _x}{\alpha _x} \frac{|\alpha _x|}{|\beta _x|} = \frac{\beta _x}{\alpha _x}\sqrt{\frac{P_{x,0}}{P_{x,1}}}, \end{aligned}$$

which is identical to Eq. (5). The physical implication of the above relation is that the probabilities $P_{x,0}$ and $P_{x,1}$ in the computational basis of the pointer give the relative *magnitudes* of $\alpha _x$ and $\beta _x$, whereas the probabilities $P_{x,\pm }$ and $P_{x,L/R}$ give the relative *phases* between them.

### Dangerous cases

There are three dangerous cases where the wavefunction extraction scheme in Eq. (13) may not give a unique solution:

(i) In the first case, $\hat{P}$ is compatible with the computational basis, $\{\mathinner {|{\textstyle x}\rangle }\}$ ($[\hat{X},\hat{P}]=0$). Then, $\mathinner {|{\textstyle x}\rangle }$ are essentially eigenstates of $\hat{P}$, and the pointer state upon the measurement of $\hat{X}$ becomes $\mathinner {|{\textstyle \phi _x}\rangle } = \psi _x(\mathinner {|{\textstyle 0}\rangle }+\mathinner {|{\textstyle 1}\rangle }e^{i\theta x}).$ Because $\psi _x$ is an overall factor, it cannot be extracted.
(ii) In the second case, the unitary $\hat{V}$ is block diagonal (possibly after simultaneous permutations of rows and columns) in a given basis. Suppose that $\hat{V}=\hat{V}^{(1)}\oplus \hat{V}^{(2)}$ with $\hat{V}^{(1)}$ and $\hat{V}^{(2)}$ operating on orthogonal subspaces $\mathscr {H}^{(1)}$ and $\mathscr {H}^{(2)}$, respectively, of $\mathscr {H}^{(1)}\oplus \mathscr {H}^{(2)}=\mathscr {H}$. Accordingly, any state $\mathinner {|{\textstyle \psi }\rangle }$ is decomposed into $\mathinner {|{\textstyle \psi }\rangle }= \mathinner {|{\textstyle \psi ^{(1)}}\rangle }\oplus \mathinner {|{\textstyle \psi ^{(2)}}\rangle }$. Upon the measurement of $\hat{X}$, the pointer is cast to
  22 $$\begin{aligned} \mathinner {|{\textstyle \phi _x}\rangle } =\mathinner {|{\textstyle 0}\rangle }\psi _x^{(\nu )}+\mathinner {|{\textstyle 1}\rangle }\sum _y\hat{V}_{xy}^{(\nu )}\psi _x^{(\nu )} \end{aligned}$$
  for $\mathinner {|{\textstyle x}\rangle }\in \mathscr {H}^{(\nu )}$ ($\nu =1,2$). Therefore, in this case, one can assess $\psi _x^{(\nu )}/\psi _0^{(\nu )}$ by applying the wavefunction extraction scheme (6) for each sector $\nu$. However, it is impossible to extract the phase relations between different sectors.
(iii) The third case is a special case where $\mathinner {|{\textstyle \psi }\rangle }$ happens to be an eigenstate of $\hat{P}$ (i.e., $\hat{V}$) belonging to a *degenerate* eigenvalue *p*. Suppose that the pointer is in the state $\mathinner {|{\textstyle \phi _x}\rangle }=\psi _x(\mathinner {|{\textstyle 0}\rangle }+\mathinner {|{\textstyle 1}\rangle }e^{i\theta p})$ after the measurement of $\hat{X}$ on the system. The two-state tomography can successfully extract the relative phase factor $e^{i\theta p}$, and hence *p*. If *p* is non-degenerate, the eigenvalue itself uniquely identifies $\mathinner {|{\textstyle \psi }\rangle }$ as its eigenstate. However, it is impossible if *p* is degenerate. Fortunately, this special case can be discerned experimentally because $\varphi _x$ is independent of *x*, and $P_{x,0}=P_{x,1}$ for all *x*.

The first two cases can be avoided simply by properly choosing either the coupling operator $\hat{P}$ or the computational basis $\mathinner {|{\textstyle x}\rangle }$.

### Iterative ML algorithm

Here, we detail the maximization of the likelihood function over the entire Hilbert space. Because of the normalization constraint, it is more convenient to maximize

23 $$\begin{aligned} \log \mathscr {L}[\psi ] - \lambda \sum _x|\psi _x|^2, \end{aligned}$$

where $\lambda$ is the Lagrange multiplier. Suppose that the system was initially in a definite state $\mathinner {|{\textstyle y}\rangle }$ and went through the unitary interaction $\hat{U}_\mathrm {int}$ with the pointer. Let $\mathinner {|{\textstyle \phi _{xy}}\rangle }$ be the pointer state upon the measurement outcome *x* on the system. Explicitly, it can be expressed as

24 $$\begin{aligned} \mathinner {|{\textstyle \phi _{xy}}\rangle } := \mathinner {|{\textstyle 0}\rangle }\delta _{xy} + \mathinner {|{\textstyle 1}\rangle }V_{xy}. \end{aligned}$$

The pointer state $\mathinner {|{\textstyle \phi _x}\rangle }$ resulting from the general initial state $\mathinner {|{\textstyle \psi }\rangle }$ of the system is related to $\mathinner {|{\textstyle \phi _{xy}}\rangle }$ by $\mathinner {|{\textstyle \phi _x}\rangle } = \sum _y\mathinner {|{\textstyle \phi _{xy}}\rangle }\psi _y$.

In terms of $\mathinner {|{\textstyle \phi _{xy}}\rangle }$, the joint probability can be expressed as

25 $$\begin{aligned} P_{x,m} = \mathinner {\langle {\textstyle \phi _x|\hat{\Pi }_m|\phi _x}\rangle } = \sum _{yz}\mathinner {\langle {\textstyle m|\phi _{xy}}\rangle }^*\psi _y^*\mathinner {\langle {\textstyle m|\phi _{xz}}\rangle }\psi _z. \end{aligned}$$

For later use, it should be noted that its derivative with respect to $\psi _x$ has the form

26 $$\begin{aligned} \frac{\partial P_{x,m}}{\partial \psi _y^*} = \sum _{z}\mathinner {\langle {\textstyle m|\phi _{xy}}\rangle }^*\mathinner {\langle {\textstyle m|\phi _{xz}}\rangle }\psi _z. \end{aligned}$$

Then, the extremal equation for the maximization problem (23) is given by

27 $$\begin{aligned} \frac{\log \mathscr {L}}{\partial \psi _y^*} = \sum _{x}\sum _{m\in \mathscr {M}}\frac{F_{x,m}}{P_{x,m}} \frac{\partial P_{x,m}}{\partial \psi _y^*} = \sum _{xz}\sum _{m\in \mathscr {M}} \mathinner {\langle {\textstyle \phi _{xy}|m}\rangle } \frac{F_{x,m}}{P_{x,m}} \mathinner {\langle {\textstyle m|\phi _{xz}}\rangle }\psi _z = \psi _y. \end{aligned}$$

We define an *x*-dependent operator $\hat{R}_x$ on the pointer by

28 $$\begin{aligned} \hat{R}_x := \sum _{m\in \mathscr {M}} \frac{F_{x,m}}{P_{x,m}} \mathinner {|{\textstyle m}\rangle }\mathinner {\langle {\textstyle m}|} = \sum _{m\in \mathscr {M}} \frac{F_{x,m}}{P_{x,m}}\hat{\Pi }_m. \end{aligned}$$

Then, the extremal equation (27) is

29 $$\begin{aligned} \sum _{xz}\mathinner {\langle {\textstyle \phi _{xy}}|}\hat{R}_x\mathinner {|{\textstyle \phi _{xz}}\rangle }\psi _z = \psi _y. \end{aligned}$$

Putting (24) into the above equation, we obtain

30 $$\begin{aligned} \sum _{xz}\left( \delta _{yx}\mathinner {\langle {\textstyle 0}|}+V_{yx}^\dag \mathinner {\langle {\textstyle 1}|}\right) \hat{R}_x \left( \mathinner {|{\textstyle 0}\rangle }\delta _{xz}+\mathinner {|{\textstyle 1}\rangle }V_{xz}\right) \psi _z = \psi _y, \end{aligned}$$

which is identical to the matrix equation (13).

### Matrix product states and operators

Consider a system of *n* particles, each of which has Hilbert space dimension *d*. We denote the computational basis state $\mathinner {|{\textstyle x}\rangle }$ for $x=0,1,\ldots ,d^n-1$ as $\mathinner {|{\textstyle x}\rangle } = \mathinner {|{\textstyle x_1}\rangle }\otimes \mathinner {|{\textstyle x_2}\rangle }\otimes \cdots \mathinner {|{\textstyle x_n}\rangle }$, where $x_j$ are the base *d* digits in *x*, $x = x_1 + x_2 d + \cdots + x_n d^{n-1}$.

An open boundary matrix product state (MPS)^23,24^ is represented by

31 $$\begin{aligned} \mathinner {|{\textstyle \eta }\rangle }= \sum _{x}\mathinner {|{\textstyle x_1}\rangle }\otimes \mathinner {|{\textstyle x_2}\rangle }\otimes \cdots \otimes \mathinner {|{\textstyle x_n}\rangle } A_1^{x_1}A_2^{x_2}\cdots A_n^{x_n}, \end{aligned}$$

where $A_j^{x_j}$ are the $D_j\times D_{j+1}$ complex matrices, depending on the local state $x_j$, and $D_1=D_{N+1}=1$. Similarly, an open boundary matrix product operator (MPO) takes the form

32 $$\begin{aligned} \hat{O}= \sum _{\mu _1=1}^{d^2}\sum _{\mu _2}\cdots \sum _{\mu _n} \hat{\tau }_1^{\mu _1}\otimes \hat{\tau }_2^{\mu _2}\otimes \cdots \otimes \hat{\tau }_n^{\mu _n}\, B_1^{\mu _1} B_2^{\mu _1}\cdots B_n^{\mu _1}, \end{aligned}$$

where $\hat{\tau }_j^{\mu _j}$ are the basis operators of the Hilbert space of all linear operators acting on particle *j*; and $B_j^{\mu _j}$ are $D_j'\otimes D_{j+1}'$ complex matrices ($D_1'=D_{n+1}'=1$).

One can observe that the conditional operator $\sum _x\mathinner {|{\textstyle x}\rangle }\mathinner {\langle {\textstyle x}|}\otimes \hat{R}_x[\psi ^{(k)}]$ is an MPO with a finite bond dimension provided that the state $\mathinner {|{\textstyle \psi ^{(k)}}\rangle }$ is an MPS with a finite bond dimension. Because an MPS has finite correlations, the probabilities $P_{x_1\dots x_n,m}$ are factorized as they are statistically independent of the uncorrelated parts^23,24^; we recall the base-*d* digits representation of *x*. This is also the case for the experimental observed frequencies $F_{x_1\dots x_n,m}$. Therefore, the conditional operator is an MPO with a finite bond dimension.

## Data Availability

The source code that support the findings of this study are available from the author upon reasonable request.
